# Change in mRNA Expression after Atenolol, a Beta-adrenergic Receptor Antagonist and Association with Pharmacological Response

**DOI:** 10.1111/j.1753-5174.2009.00020.x

**Published:** 2009-09

**Authors:** Utkarsh Kohli, Britney L Grayson, Thomas M Aune, Laxmi V Ghimire, Daniel Kurnik, C Michael Stein

**Affiliations:** * Department of Medicine, Division of Clinical PharmacologyNashville, TN, USA; † Rheumatology, Vanderbilt UniversityNashville, TN, USA; ‡ Department of Medicine, Division of Clinical Pharmacology, Chaim Sheba Medical Center, Ramat Gan, and Sackler School of Medicine, Tel Aviv UniversityTel Aviv, Israel

**Keywords:** Atenolol, mRNA expression, Microarray

## Abstract

**Aims:**

Genetic determinants of variability in response to β-blockers are poorly characterized. We defined changes in mRNA expression after a β-blocker to identify novel genes that could affect response and correlated these with inhibition of exercise-induced tachycardia, a measure of β-blocker sensitivity.

**Methods:**

Nine subjects exercised before and after a single oral dose of 25mg atenolol and mRNA gene expression was measured using an Affymetrix GeneChip Human Gene 1.0 ST Array. The area under the heart rate-exercise intensity curve (AUC) was calculated for each subject; the difference between post- and pre-atenolol AUCs (Δ AUC), a measure of β-blocker response, was correlated with the fold-change in mRNA expression of the genes that changed more than 1.3-fold.

**Results:**

Fifty genes showed more than 1.3-fold increase in expression; 9 of these reached statistical significance (*P* < 0.05). Thirty-six genes had more than 1.3-fold decrease in expression after atenolol; 6 of these reached statistical significance (*P* < 0.05). Change in mRNA expression of *FGFBP2* and Probeset ID 8118979 was significantly correlated with atenolol response (*P* = 0.03 and 0.02, respectively).

**Conclusion:**

The expression of several genes not previously identified as part of the adrenergic signaling pathway changed in response to a single oral dose of atenolol. Variation in these genes could contribute to unexplained differences in response to β-blockers.

## Introduction

Beta-blockers are frequently prescribed to treat ischemic heart disease, heart failure, hypertension, and arrhythmias [1–5]. They block the effects of agonists acting on β-adrenergic receptors (ARs) and influence downstream signaling pathways. There are substantial interindividual and ethnic differences in response to β-blockers that are partly accounted for by variation in the genes encoding the β_1_-AR (*ADRB1*) and mediators of downstream signaling pathways [6,7]. The relationship between variability in *ADRB1* and other candidate genes and variability in response to β-blockers has been extensively evaluated [6,8], but much of the variability remains unexplained.

Another approach to identifying the mechanisms underlying interindividual variability in response would be to identify additional candidate genes by examining the changes in messenger RNA (mRNA) that occur after exposure to a β-blocker. There is no information regarding such an approach.

Therefore, we carried out this exploratory study to identify novel genes that may regulate response to a β-blocker by measuring changes in mRNA expression after oral administration of a β-blocker (atenolol) to subjects who underwent an exercise test to determine β-blocker sensitivity. We also studied the correlation between change in mRNA expression after atenolol administration and β-blocker sensitivity, assessed by attenuation of exercise-induced tachycardia [9].

## Methods

### Subjects

This study was approved by the Institutional Review Board of Vanderbilt University Medical Centre, Nashville, TN, and all subjects gave written informed consent. We enrolled 9 unrelated subjects; one subject was excluded from analysis because the post-treatment sample could not be hybridized. Subjects were eligible to participate if they were between 18–40 years of age and had no clinically significant abnormality based on medical history, physical examination, electrocardiogram, and routine laboratory testing. Subjects reported their ethnicity and that of their parents and grandparents using checkboxes to choose among “Caucasian”, “African–American”, “Hispanic”, “Chinese”, “Japanese”, and “other” (the latter to be specified). Multiple choices were permitted. A patient was assigned to an ethnic group when both parents and at least three out of four grandparents were of the same ethnicity. Patients were free of medications and dietary supplements for at least 1 week and received a controlled alcohol-free and caffeine-free diet (providing 150 mmol of sodium, 70 mmol of potassium, and 600 mmol of calcium daily) for 5 days before the study.

### Protocol

Details of the exercise protocol have been described in detail elsewhere [6]. Briefly, after an overnight fast a 20 G intravenous cannula was inserted into an antecubital arm vein for blood sampling and after 30 minutes of supine rest, a baseline blood sample was drawn for mRNA analysis into two PAXgene Blood mRNA Tubes (PreAnalytiX/Qiagen Inc., Valencia, CA), incubated at room temperature for 2 hours, and then stored at −20°C. Then, subjects exercised on an electronically braked supine bicycle ergometer at sequentially increasing workloads of 25, 50, and 75 watts for 2 minutes each. Then, 10 minutes after completion of exercise, subjects swallowed a 25 mg tablet of atenolol. A second blood sample for mRNA expression was collected 2.5 hours after atenolol (to coincide with peak atenolol concentrations) and immediately after the blood draw a second exercise test was performed as per previously described protocol.

### mRNA

Total mRNA was extracted from whole blood using PAXgene Blood mRNA Kit (Qiagen, Valencia, CA) and then subjected to DNase treatment according to the manufacturer's instructions (Qiagen, Valencia, CA). The mRNA were assessed for concentration by spectrophotometry and integrity using the Agilent Bioanalyzer (Agilent Technologies, Palo Alto, CA), and then stored at −20°C.

### Microarray

Following quality control, the mRNA was prepared for microarray analysis using the GeneChip Whole Transcript (WT) Sense Target Labeling Assay protocol (Affymetrix Inc, Santa Clara, CA). Briefly, a total of 100ng of total mRNA was reverse transcribed to cDNA T7-random primers followed by second-strand synthesis. The double-stranded cDNA was then used as template in an *in vitro* transcription reaction followed by cDNA synthesis, fragmentation of the single stranded cDNA and labeling through a terminal deoxy-transferase reaction. The biotinylated cDNA (5 µg) was fragmented and hybridized to an Affymetrix GeneChip Human Gene 1.0 ST Array (Affymetrix Inc, Santa Clara, CA).

### Data Analysis

Following scanning, CEL files were imported into Partek Genomic Suitev6.4 (Partek Inc, St Louis, MO) and robust multi-chip average (RMA) normalized. A paired-sample t-test was performed between the pre-treatment and post-treatment groups. A 1.3-fold change in gene expression was considered potentially significant [10].

Demographic data are expressed as mean ± standard deviation (SD). We used two-sample Wilcoxon rank-sum (Mann-Whitney) test to compare outcomes before and after atenolol. A response-feature approach was used to model multiple heart rate measurements in the same subjects [11]. In these analyses, the response feature was the area under the heart rate-exercise intensity curve (AUC) for each subject. The difference between post- and pre-atenolol heart rate AUCs (Δ AUC) was calculated to determine the response to atenolol and this was correlated with the fold-change in mRNA expression of the genes that were significantly upregulated and downregulated (±1.3-fold) using a non-parametric measure of correlation (Spearman's rank correlation coefficient). Analyses were performed using the statistical software STATA v.10.0 (StataCorp, College Station, TX) and Partek (Partek Inc, St Louis, MO).

## Results

### Subjects

The demographic characteristics of the study subjects (n = 8) are described in Table 1.

**Table 1:** Demographic characteristics

Characteristic (N = 8)	Mean ± SD
Age (years)	28.3 ± 6.3
Sex (Male/Female)	5/3
Ethnicity: Caucasian/African American/Hispanic/ Asian	2/1/1/4
Weight (kg)	70.5 ± 12.0
Height (m)	1.72 ± 0.08

### Atenolol Effect

Atenolol significantly reduced the resting heart rate (mean reduction = 6.5 ± 6.8 beats/minute; *P* = 0.02), and heart rate at all the exercise stages (mean reduction = 13 ± 10.2, 13.1 ± 9.9, 19.0 ± 9.5 beats/minute at 25, 50, and 75W of exercise, respectively; all *P* < 0.02). Heart rate-AUC was also significantly reduced (mean reduction = 1050 ± 658 beats/minute.watt; *P* = 0.02).

### Microarray

**Figure 1:**
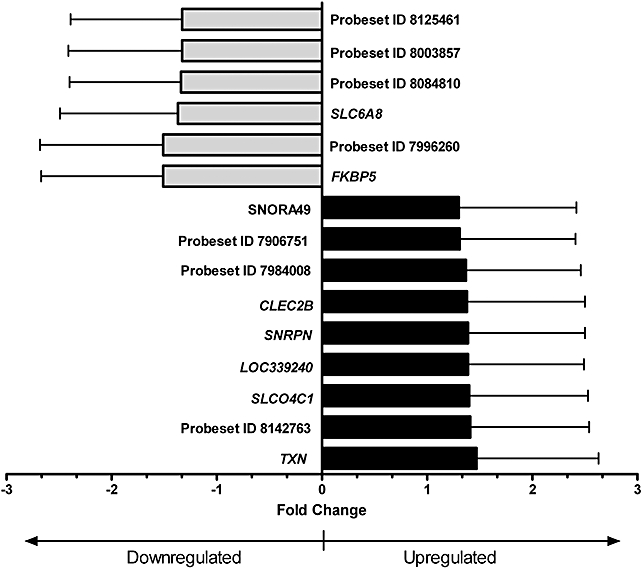
Significantly upregulated and downregulated genes. Error bars represent standard error.

There were 50 genes upregulated more that 1.3-fold (Table 2). Change in mRNA expression for 9 of these genes (*TXN*, *SLCO4C1, LOC339240, SNRPN, CLEC2B, SNORA49* and Probeset IDs 8142763, 7984008, 7906751) reached statistical significance (*P* < 0.05) (Figure 1). A range of other genes including WD repeat domain 74, small cajal body-specific mRNA 7, killer cell lectin-like receptor subfamily F, S100 calcium binding protein A12, and fibroblast growth factor binding protein 2 were also upregulated. Change in mRNA expression for fibroblast growth factor binding protein 2 (*FGFBP2*) correlated significantly with atenolol effect (Δ AUC) (Spearman coefficient = −0.76; *P* = 0.03).

**Table 2:** Genes upregulated more than 1.3-fold

Gene symbol	Gene title	Ref seq ID	Function	Fold change ± SE	*P* value*	Spearman's rho	*P* value**
*RPL34*	Ribosomal Protein L34 (4q25)	CR542242	Ribosomal protein: component of the 60S subunit.	1.52 ± 1.39	0.25	−0.45	0.26
*WDR74*	WD repeat domain 74 (11q12.3)	AK292330		1.50 ± 1.2	0.06	0.05	0.91
*SCARNA7*	Small Cajal body-specific RNA 7 (3q25.22)	NR_003001		1.48 ± 1.34	0.22	−0.31	0.45
*SNRPN*	Small Nuclear Ribonucleoprotein polypeptide N (15q11.2)	NR_003323	Role in pre-mRNA processing, possibly tissue-specific alternative splicing events.	1.47 ± 1.19	0.07	−0.24	0.56
*TXN*	Thioredoxin (9q31)	NM_003329		1.47 ± 1.16	0.04*	−0.05	0.91
*SNRPN*	Small Nuclear Ribonucleoprotein Polypeptide N (15q11.2)	NR_003318	Role in pre-mRNA processing, possibly tissue-specific alternative splicing events.	1.47 ± 1.22	0.09	−0.48	0.22
*RPS3A*	Ribosomal Protein S3A (4q31.2-q31.3)	NM_001006	Ribosomal protein: component of the 40S subunit.	1.45 ± 1.31	0.21	−0.24	0.57
hCG_1983332	hCG1983332 (7q11.21)	NR_003536		1.45 ± 1.18	0.06	−0.39	0.33
8144569 (Probeset ID)				1.45 ± 1.22	0.10	0.28	0.49
*RPL26*	Ribosomal Protein L26 (17p13)	NM_000987	Ribosomal protein: component of the 60S subunit.	1.45 ± 1.43	0.33	−0.31	0.45
*KLRF1*	Killer Cell Lectin-like Receptor Subfamily F, member 1 (12p13.31)	NM_016523		1.44 ± 1.18	0.07	−0.52	0.18
8142763 (Probeset ID)				1.41 ± 1.13	0.03*	0.28	0.49
*RPL9*	Ribosomal Protein L9 (4p13)	NM_001024921	Ribosomal protein: component of the 60S subunit.	1.41 ± 1.31	0.24	−0.19	0.65
*RPL9*	Ribosomal Protein L9 (4p13)	NM_000661	Ribosomal protein: component of the 60S subunit.	1.41 ± 1.31	0.24	−0.14	0.73
*S100A12*	S100 Calcium Binding Protein A12 (1q21)	NM_005621	Involved in the regulation of cell cycle progression, differentiation and calcium-dependent signal transduction pathways.	1.41 ± 1.25	0.16	0.02	0.95
*SNRPN*	Small Nuclear Ribonucleoprotein Polypeptide N (15q11.2)	NR_003316	Pre-mRNA processing, possibly tissue-specific alternative splicing events.	1.41 ± 1.19	0.10	−0.36	0.38
*LSM3*	LSM3 Homolog, U6 Small Nuclear mRNA associated (S. cerevisia)	NM_014463	Pre-mRNA splicing.	1.40 ± 1.22	0.13	−0.36	0.38
7984008 (Probeset ID)				1.40 ± 1.13	0.03*	−0.26	0.53
*FGFBP2*	Fibroblast Growth Factor Binding Protein 2 (4p16)	NM_031950	Secreted by cytotoxic lymphocytes and involved in cytotoxic lymphocyte-mediated immunity.	1.40 ± 1.16	0.06	−0.76	0.03**
hCG_1787519	Similar to Large Subunit Ribosomal Protein L36a	NM_001101383		1.39 ± 1.36	0.31	−0.31	0.45
*SCARNA10*	Small Cajal Body-Specific RNA 10 (12p13.31)	NR_004387		1.39 ± 1.16	0.06	−0.48	0.23
*SLCO4C1*	Solute Carrier Organic Anion Transporter Family, member (5q21.2)	NM_180991		1.39 ± 1.10	0.01*	−0.14	0.73
*LOC*339240	Keratin Pseudogene (17p11.2)	ENST00000332088		1.39 ± 1.11	0.02*	−0.31	0.45
*S100A8*	S100 Calcium Binding Protein A8 (1q21)	NM_002964	Involved in the regulation of cell cycle progression and differentiation.	1.39 ± 1.26	0.20	−0.38	0.35
*KLRB1*	Killer Cell Lectin-Like Receptor Subfamily B, member 1 (12p13.31)	NM_002258	Type II membrane protein.	1.39 ± 1.20	0.11	−0.45	0.26
*SNRPN*	Small Nuclear Ribonucleoprotein Polypeptide N (15q11.2)	NR_003330	Pre-mRNA processing, possibly tissue-specific alternative splicing events. 5' UTR: imprinting center.	1.38 ± 1.12	0.02*	−0.52	0.17
*CLEC2B*	C-type Lectin Domain Family 2, member B (12p13.31)	NM_005127	Affects cell adhesion, cell-cell signaling, glycoprotein turnover, inflammation and immune response.	1.37 ± 1.09	0.007*	−0.04	0.91
8102728 (Probeset ID)				1.37 ± 1.31	0.28	−0.57	0.14
C15orf15	Chromosome 15 Open Reading Frame 15 (15q21)	NM_016304	Protein shares a low level of sequence similarity with human ribosomal protein L24.	1.37 ± 1.19	0.11	−0.33	0.42
*TINP1*	TGF Beta-Inducible Nuclear Protein 1 (5q13.3)	NM_014886		1.36 ± 1.21	0.16	−0.55	0.16
*SNORD28*	Small Nucleolar RNA, C/D box 28 (11q13)	NR_002562		1.35 ± 1.27	0.25	−0.12	0.78
8065325 (Probeset ID)				1.35 ± 1.20	0.14	−0.19	0.65
*GZMA*	Granzyme A (Granzyme 1, Cytotoxic T-Lymphocyte-Associated) (5q11.2)	NM_006144	T cell- and natural killer cell-specific serine protease.	1.35 ± 1.15	0.07	−0.55	0.16
*RPS17*	Ribosomal Protein S17 (15q)	NM_001021	Ribosomal protein: component of the 40S subunit.	1.34 ± 1.31	0.31	−0.24	0.57
*RPL36A*	Ribosomal Protein L36a (Xq22.1)	NM_021029	Ribosomal protein: component of the 40S subunit.	1.33 ± 1.18	0.32	−0.40	0.31
hCG_2004593	Ribosomal Protein L17-Like (15q23)	NM_001093733	Pseudogene derived from the RPL17 gene.	1.33 ± 1.18	0.30	−0.24	0.57
*EVI2A*	Ecotropic Viral Integration Site 2A (17q11.2)	NM_001003927		1.33 ± 1.14	0.12	−0.12	0.78
*SNRPN*	Small Nuclear Ribonucleoprotein Polypeptide N (15q11.2)	NR_003320	Pre-mRNA processing, possibly tissue-specific alternative splicing events.	1.33 ± 1.14	0.07	−0.33	0.41
*SNRPN*	Small Nuclear Ribonucleoprotein Polypeptide N (15q11.2)	NR_003338	Pre-mRNA processing, possibly tissue-specific alternative splicing events.	1.33 ± 1.13	0.07	−0.48	0.23
8107940 (Probeset ID)				1.31 ± 1.13	0.07	−0.31	0.45
*LOC91664*	Hypothetical Protein BC007307 (19q13.41)	ENST00000357015		1.31 ± 1.16	0.11	0.26	0.53
7906751 (Probeset ID)				1.31 ± 1.10	0.03*	0.40	0.32
*RPL7*	Ribosomal Protein L7 (8q21.11)	NM_000971	Ribosomal protein: component of the 60S subunit.	1.31 ± 1.16	0.38	−0.31	0.45
*SF3B14*	Splicing Factor 3B, 14 kDa subunit (2p23.3)	NM_016047	14 kDa protein subunit of the splicing factor 3b complex.	1.31 ± 1.33	0.12	0.02	0.95
8103222 (Probeset ID)				1.31 ± 1.30	0.35	−0.24	0.57
*RPL24*	Ribosomal Protein L24 (3q12)	NM_000986	Ribosomal protein: component of the 60S subunit.	1.30 ± 1.21	0.20	−0.43	0.29
*RPL17*	Ribosomal Protein L17 (18q21)	NM_001035006	Ribosomal protein: component of the 60S subunit.	1.30 ± 1.28	0.31	−0.24	0.57
*RPL36AL*	Ribosomal Protein L36a-Like (14q21)	NM_001001	Ribosomal protein: component of the 60S subunit.	1.30 ± 1.17	0.14	−0.31	0.45
*SNORA49*	Small Nucleolar RNA, H/ACA box 49 (12q24.33)	NR_002979		1.30 ± 1.12	0.05*	−0.09	0.82
8130370 (Probeset ID)				1.30 ± 1.17	0.13	0.59	0.11

* *P* value represents the change in mRNA comparing before and after atenolol;

** *P* value represents the significance of Spearman's correlation coefficient examining the association between fold-change in mRNA expression and attenuation of exercise-induced tachycardia by atenolol.

Thirty-six genes were downregulated at least 1.3-fold after atenolol administration (Table 3); the decrease in mRNA expression for 6 of these genes (*FKBP5, SLC6A8* and Probeset IDs 7996260, 8084810, 8003857, 8125461) reached statistical significance (*P* < 0.05) (Figure 1). Change in mRNA expression of Probeset ID 8118979 after atenolol administration was significantly correlated with the decrease in heart rate AUC (Spearman correlation coefficient = 0.77; *P* = 0.02). Change in mRNA expression for none of the other upregulated or downregulated genes correlated significantly with attenuation of exercise induced tachycardia.

**Table 3:** Genes downregulated more than 1.3-fold

Gene symbol	Gene title	Ref seq ID	Function	Fold change ± SE	*P* value*	Spearman's Rho	*P* value**
*SLC4A1*	Solute Carrier Family 4, Anion Exchanger, Member 1 (17q21.31)	NM_000342	Erythrocyte chloride/bicarbonate exchanger involved in carbon dioxide transport.	−1.53 ± 1.29	0.14	−0.12	0.78
*HBZ*	Hemoglobin, Zeta (16p13.3)	NM_005332	Alpha-like hemoglobin. Synthesized in the yolk sac of the early embryo.	−1.53 ± 1.26	0.11	−0.53	0.18
*FKBP5*	FK506 Binding Protein 5 (6p21.3-p21.2)	NM_004117	Role in immunoregulation and calcineurin inhibition.	−1.51 ± 1.16	0.03*	0.17	0.69
7996260 (Probeset ID)				−1.51 ± 1.17	0.04*	−0.29	0.49
8169638 (Probeset ID)				−1.48 ± 1.24	0.11	−0.43	0.28
*TRIM58*	Tripartite Motif-Containing 58 (1q44)	NM_015431		−1.47 ± 1.22	0.09	−0.24	0.56
*SELENBP1*	Selenium Binding Protein 1 (1q21-q22)	NM_003944	Selenium-binding protein family.	−1.45 ± 1.25	0.14	−0.2	0.64
*ALAS2*	Delta-Aminolevulinate Synthase 2 (Xp11.21)	NM_000032	Erythroid-specific mitochondrially located enzyme.	−1.41 ± 1.26	0.18	−0.19	0.64
*GMPR*	Guanosine Monophosphate Reductase (6p23)	NM_006877	Catalyzes the irreversible NADPH-dependent reductive deamination of guanosine monophosphate (GMP) to inosine monophosphate (IMP).	−1.40 ± 1.20	0.11	−0.19	0.64
*EPB49*	Erythrocyte Membrane Protein band 4.9 (dematin) (8p21.1)	NM_001978		−1.40 ± 1.27	0.20	−0.21	0.61
*SLC38A5*	Solute Carrier Family 38, member 5 (Xp11.23)	NM_033518	Mediates Na(+)-coupled transport of neutral amino acids.	−1.40 ± 1.25	0.18	−0.43	0.28
*ALS2CR2*	Amyotrophic Lateral Sclerosis 2 (juvenile) Chromosome region (2q33.1)	NM_018571		−1.38 ± 1.28	0.23	−0.2	0.64
*SNCA*	Alpha Synuclein (non A4 component of amyloid precursor) (4q22.1)	NM_000345	Inhibits phospholipase D2.	−1.38 ± 1.26	0.20	−0.19	0.65
*SLC6A8*	Solute Carrier Family 6 (neurotransmitter transporter) (Xq28)	NM_005629	Transports creatine into and out of cells.	−1.37 ± 1.12	0.03*	−0.38	0.34
*OR2W3*	Olfactory Receptor, Family 2, Subfamily W, Member 3 (1q44)	NM_001001957		−1.36 ± 1.17	0.09	−0.41	0.31
C16orf35	Chromosome 16 Open Reading Frame 35 (6p13.3)	NM_001077350		−1.36 ± 1.22	0.17	−0.55	0.15
8118979 (Probeset ID)				−1.36 ± 1.21	0.15	0.77	0.02**
*GYPC*	Glycophorin C (Gerbich blood group) (2q14-q21)	NM_002101	An integral membrane glycoprotein.	−1.35 ± 1.24	0.21	−0.40	0.32
*CSDA*	Cold Shock Domain Protein A (12p13.1)	NM_003651		−1.35 ± 1.23	0.19	−0.12	0.78
8149953 (Probeset ID)				−1.34 ± 1.19	0.14	0.55	0.16
*SLC25A39*	Solute Carrier Family 25, Member 39 (17q12)	NM_016016	Belongs to the SLC25 family of mitochondrial carrier proteins.	−1.34 ± 1.24	0.21	−0.34	0.41
*WDR40A*	WD Repeat Domain 40A (9p13.3)	NM_015397		−1.34 ± 1.22	0.19	−0.19	0.65
*BCL2L1*	BCL2-like 1 (20q11.21)	NM_138578	Anti- or pro-apoptotic regulator. Involved in a wide variety of cellular activities.	−1.34 ± 1.21	0.18	−0.19	0.65
8084810 (Probeset ID)				−1.34 ± 1.06	0.002*	0.53	0.18
8003857 (Probeset ID)				−1.33 ± 1.08	0.008*	0.58	0.13
8125461 (Probeset ID)				−1.33 ± 1.06	0.002*	0.38	0.34
*FAM46C*	Family with Sequence Similarity 46, Member C (1p12)	NM_017709		−1.33 ± 1.20	0.16	−0.2	0.64
7961418 (Probeset ID)				−1.33 ± 1.18	0.13	−0.14	0.73
8115443 (Probeset ID)				−1.33 ± 1.25	0.25	0.13	0.76
7956269 (Probeset ID)				−1.33 ± 1.34	0.37	0.62	0.1
*TMEM63B*	Transmembrane Protein 63B (6p21.1)	NM_018426		−1.32 ± 1.21	0.18	−0.34	0.41
7989193 (Probeset ID)				−1.32 ± 1.16	0.11	0.31	0.45
*PDZK1IP1*	PDZK1 Interacting Protein 1 (1p33)	NM_005764		−1.32 ± 1.14	0.08	0.05	0.90
*GYPA*	Glycophorin A (MNS blood group) (4q28.2-q31.1)	NM_002099	Sialoglycoproteins of the human erythrocyte membrane. The antigenic determinants for the MN and Ss blood groups.	−1.31 ± 1.30	0.32	−0.02	0.95
*TMOD1*	Tropomodulin 1 (9q22.3)	NM_003275		−1.31 ± 1.21	0.20	−0.19	0.65
*RBM38*	mRNA Binding Motif Protein 38 (20q13.31)	NM_017495		−1.30 ± 1.15	0.10	−0.05	0.91

* *P* value represents the change in mRNA comparing before and after atenolol;

** *P* value represents the significance of Spearman's correlation coefficient examining the association between fold-change in mRNA expression and attenuation of exercise-induced tachycardia by atenolol.

## Discussion

There is no information about the effect of a β-blocker on mRNA expression, thus our finding that several genes are upregulated or downregulated are of interest. There are 50 genes upregulated more than 1.3-fold, and change in mRNA expression for 9 of these genes is statistically significant (*P* < 0.05). Many of these genes code for ribosomal proteins, small nuclear ribonucleoprotein polypeptides and signal transduction pathways that have not previously been associated with β-blocker signaling. One of the significantly upregulated genes (*TXN*) codes for thioredoxin. Thioredoxins act as antioxidants by facilitating the reduction of other proteins by cysteine thiol-disulfide exchange [12,13]. The thioredoxins are kept in the reduced state by the flavoenzyme thioredoxin reductase, in a NADPH-dependent reaction [14]. Thioredoxin reductase activity is indirectly regulated by β_2_-ARs in human cutaneous tissue; its activity in human melanoma cells is stimulated by calcium, and calcium exchange between these cells and surrounding skin is stimulated by β_2_-ARs [15]. Another upregulated gene, *SLCO4C1*, codes for an organic anion transporter that is expressed on the basolateral membrane of renal proximal tubular cells. It transports cardiac glycosides, thyroid hormone, cAMP, and methotrexate in a sodium-independent manner [16]. However, no clear role has been identified for these significantly upregulated genes in the adrenergic signaling pathway.

Similarly, no clear role has been identified in adrenergic signaling for other upregulated genes such as WD repeat domain 74, small cajal body-specific mRNA 7, killer cell lectin-like receptor subfamily F, S100 calcium binding protein A12, fibroblast growth factor binding protein 2, solute carrier organic anion transporter family, and keratin pseudogene.

Thirty-six genes are downregulated at least 1.3-fold after atenolol administration, and the decrease in mRNA expression for 6 of these genes (*FKBP5, SLC6A8* and Probeset IDs 7996260, 8084810, 8003857, 8125461) is statistically significant. These genes can be categorized into 3 broad ontogenic groups based on the proteins that they code: transport proteins, ion channels and cytoskeletal proteins. *FKBP5* codes for a chaperone protein that has been implicated in stress related disorders [16,17]. The other downregulated genes code for a wide variety of proteins like solute carrier family 4, anion exchanger, hemoglobin zeta, tripartite motif-containing 58, and δ-aminolevulinate synthase 2. As is the case with the upregulated genes, the role of these downregulated genes in adrenergic signaling is not known

Change in mRNA expression of *FGFBP2* and Probeset ID 8118979 is correlated significantly with the β-blocker effect. The role of these 2 genes in adrenergic signaling pathway is not known. However, *FGF2* (fibroblast growth factor 2) codes for a growth factor that is needed for maturation and survival of catecholaminergic neurons, and *FGFBP* codes for a protein that binds extracellular FGF2 and enhances its activity. Chronic administration of antidepressants, which act on noradrenergic pathways, increases the expression of these two proteins [18]. Expression of *FGF2* is also increased in cardiac myocytes in response to stimulation of α_1_-adrenoceptors [19]. Thus, FGFBP is associated with the adrenergic system, but its role in cardiac β-adrenergic signaling is not established.

This study has several limitations. Subjects exercised before atenolol was administered, and the effect of atenolol on mRNA expression could potentially have been altered by the preceding exercise. However, the majority of genes that changed more than 1.3-fold were not influenced by exercise in previous studies [20–25]. Another potential limitation is that we used whole blood for analysis of mRNA expression, and changes in mRNA expression can be lower than those obtained from isolated cells [26,27]. We studied change in mRNA expression 2.5 hours after administration of a single dose of atenolol, when peak atenolol concentrations are reached. The pattern of gene expression after atenolol may vary with time, and after chronic administration of drug. However, ethnic and genetic differences in sensitivity to atenolol measured as inhibition of exercise-induced tachycardia can be detected 2.5 hours after a single dose [6]. Therefore, the gene expression profile at this time is of interest. We administered a single dose of atenolol (25 mg) to our subjects. It is possible that the change in the pattern of mRNA expression with higher doses or with multiple doses may differ. Also, we did not correct for multiple comparisons but included only those genes that were upregulated or downregulated more than 1.3 fold to limit the false discovery rate; there is little consensus on the optimum method of correction for multiple comparisons in gene expression assays [28], and the analysis should be regarded as exploratory and hypothesis-generating.

In conclusion, in this preliminary study many genes not known to be involved with adrenergic signaling were upregulated or downregulated in response to atenolol. Change in mRNA expression for 2 of these genes is significantly correlated with atenolol-mediated attenuation of exercise-induced tachycardia. Additional studies to determine the reproducibility of the findings and the effects of chronic therapy may provide novel insights into the mechanisms of actions of β-blockers.
